# Imminent Rupture of an Infected Aortic Aneurysm Presenting as Lower Back Pain in an Elderly Patient

**DOI:** 10.7759/cureus.100660

**Published:** 2026-01-03

**Authors:** Satoshi Takashima, Yu Toda, Hirohito Hirata, Tomohito Yoshihara, Masatsugu Tsukamoto, Tadatsugu Morimoto

**Affiliations:** 1 Department of Orthopaedic Surgery, Saga University, Saga, JPN

**Keywords:** aortic aneurysm, infected abdominal aortic aneurysm, lower back pain, misdiagnosis, psoas abscess, septic shock

## Abstract

An infected abdominal aortic aneurysm (IAAA) is a rare and potentially life-threatening condition that can mimic common spinal disorders, leading to delayed diagnosis. We report a case of a 63-year-old male who presented with lower back pain and left leg numbness. Initially suspected to have a spinal pathology, further investigation revealed an IAAA complicated by a psoas abscess and septic shock. Despite initial antibiotic therapy, the aneurysm ruptured, necessitating emergency surgical intervention. Unfortunately, the patient died from circulatory failure on postoperative day four. This case highlights the diagnostic challenges of IAAA, particularly in patients presenting with nonspecific back symptoms. Clinicians should maintain a high index of suspicion for IAAA in elderly patients with back pain and signs of systemic infection, and imaging should be carefully reviewed beyond musculoskeletal structures.

## Introduction

Infected abdominal aortic aneurysm (IAAA) is an uncommon but potentially fatal vascular emergency characterized by microbial infection of the aortic wall. First described by Osler in 1885, the condition represents 0.7%-3.0% of all aortic aneurysms and is associated with a high mortality rate, particularly in cases of rupture. Risk factors include advanced age, immunocompromised states, and atherosclerosis [1]. In recent years, the incidence has increased due to a rise in immunocompromised populations and the widespread use of intravascular interventions. Early diagnosis is critical for patient survival, but is frequently delayed due to nonspecific symptoms such as back pain or fever. These symptoms often mimic more common musculoskeletal conditions, such as pyogenic spondylitis, leading to misdiagnosis and inappropriate referral [2]. We present a case of IAAA that was initially misdiagnosed as a spinal disorder, emphasizing the need for heightened clinical suspicion and comprehensive imaging evaluation.

This article was previously posted to the Authorea preprint server on December 21, 2024.

## Case presentation

Case history and examination

A 63-year-old male presented to the orthopedic clinic complaining of back pain and left leg numbness over the preceding month. He had a history of diabetes mellitus, hypertension, and dyslipidemia, but had discontinued his medications without medical advice.

On examination, the patient had no neurological deficits but exhibited swelling and warmth in the left lower limb. The left dorsal foot artery was not palpable.

**Figure 1:**
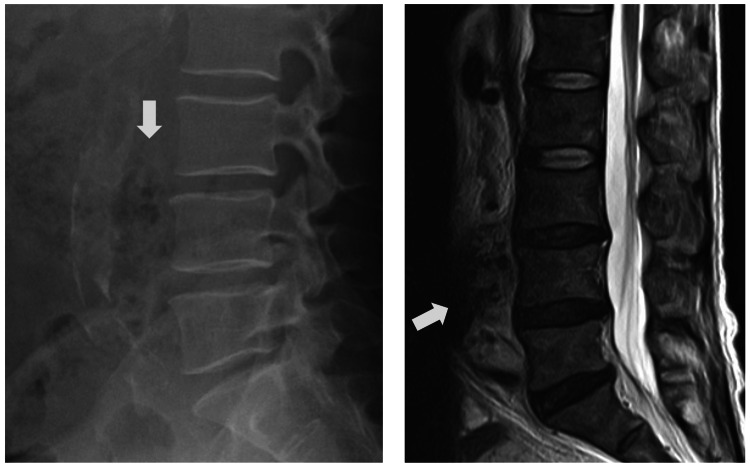
Radiographic and magnetic resonance imaging (MRI) of the lumbar spine before treatment. (A) Lumbar spine radiograph and (B) MRI showing no findings suggestive of discitis or vertebral body destructive lesions at the time of the initial examination. We observed the presence of an imminent rupture of the infected abdominal aortic aneurysm (white arrow) on reexamination.

Laboratory results showed a marked elevation in white blood cell count (26.9 × 10³/μL) and C-reactive protein level (17.96 mg/dL), along with an increased glycosylated hemoglobin (HbA1c) value (9.4%). Liver function tests were also abnormal. The presence of fever and elevated inflammatory markers suggested pyogenic spondylitis; however, imaging (radiography and magnetic resonance imaging) revealed no spinal infection (Figure 1). A liver abscess was suspected, and the patient was admitted for further evaluation.

Differential diagnosis, investigation, and treatment

**Figure 2:**
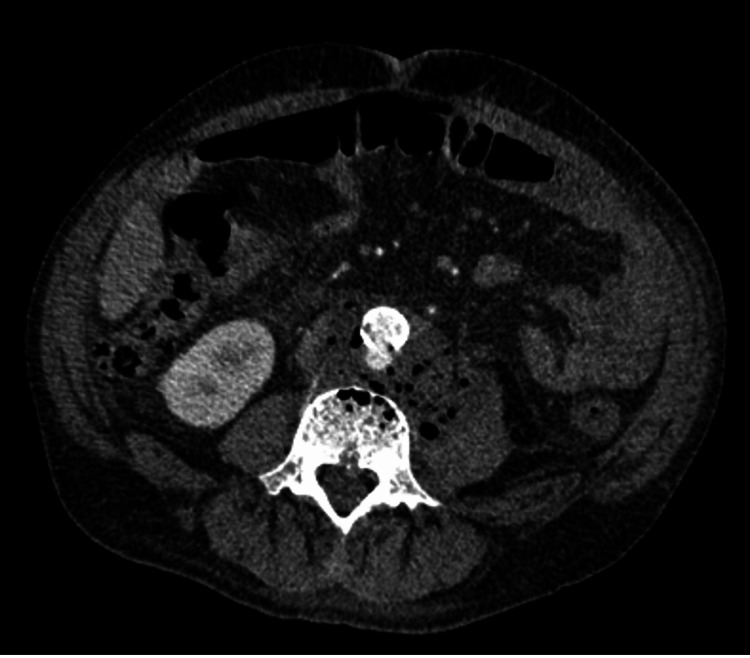
Abdominal computed tomography (CT) imaging with contrast. Contrast-enhanced abdominal CT shows a pseudoaneurysm in the descending aorta. Small, subtle gas foci were noted in the retroperitoneal space on high-resolution imaging, consistent with infection.

Contrast-enhanced abdominal computed tomography demonstrated a pseudoaneurysm of the abdominal aorta with surrounding periaortic inflammatory changes. These findings raised suspicion of an infectious aneurysm. Gas formation around the L3/4 disc space was also noted, suggesting a psoas abscess and secondary spondylodiscitis (Figure 2). A retrospective review of the initial MRI revealed missed signs of imminent IAAA rupture (Figure 1). Blood cultures identified *Escherichia coli*, confirming the diagnosis of IAAA complicated by septic shock and a psoas abscess. The patient was treated with ceftriaxone and pazufloxacin, and later transitioned to cefazolin based on susceptibility testing.

Outcome and follow-up

**Figure 3:**
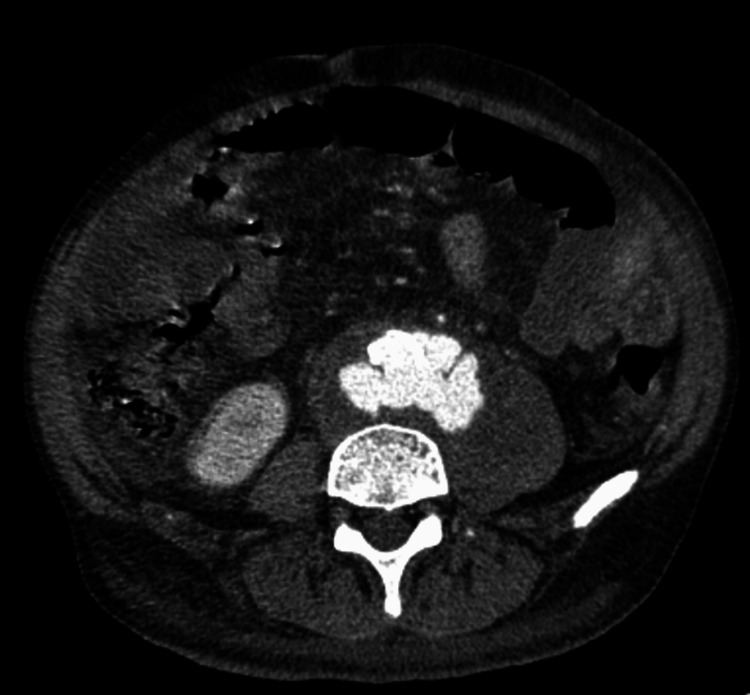
Abdominal computed tomography (CT) imaging with contrast. The pseudoaneurysm of the abdominal aorta appears markedly enlarged and in a state of imminent rupture. The abscess around the lesion and within the left iliopsoas and psoas major muscles with gas appears to be enlarged.

Although the initial antibiotic treatment stabilized the patient's condition, his fever and back pain worsened after four days. Despite ongoing therapy, computed tomography revealed a ruptured IAAA. Emergency surgery, including aorto-right external iliac bypass, femorofemoral bypass, enterectomy, and colostomy, was performed (Figure 3). The patient was managed in the intensive care unit but died on the fourth postoperative day because of circulatory failure.

## Discussion

First described by Osler in 1885, IAAA includes aneurysms arising from pre-existing aortic wall pathology [3]. Although rare (0.7%-3% of all aortic aneurysms), IAAA has a high mortality rate (5%-44%) [4] and is increasing in prevalence because of atherosclerosis and iatrogenic arterial injuries [5].

Early diagnosis is difficult because symptoms are often nonspecific [6,7]. Unlike abdominal aortic aneurysms, which typically present with abdominal pain or hemodynamic instability, IAAAs often present with back pain, prompting initial consultation in orthopedic clinics [8]. According to various authors, rupture or impending rupture of an AAA should be suspected when: (1) the patient is a middle-aged or older male with a history of an abdominal mass; (2) the pain is severe, with sudden onset and lateral abdominal components; (3) there is no tenderness in the lumbar spine or surrounding soft tissues and no neurological symptoms; (4) the iliopsoas muscle shadow is abnormal on radiography; and (5) a pulsatile mass is detected in the abdomen [9].

Similar cases have been documented. Ng and Heng [2] reported an elderly patient with back pain later diagnosed with an infected aortic aneurysm and spondylodiscitis. Similarly, Patelis et al. [8] described the diagnostic challenge in distinguishing IAAA from spinal infections, especially in the early stages. In both cases, delayed diagnosis was linked to initial misinterpretation of spinal symptoms. In our case, although MRI is generally sensitive for detecting bone marrow and disc-space infection, the initial MRI did not show definitive findings because the infection was at an early stage. However, the patient’s fever, inflammatory markers, and back pain worsened, prompting contrast-enhanced CT on the following day, which revealed psoas abscess formation and secondary spondylodiscitis. Retrospective review of the initial MRI revealed subtle soft-tissue signal changes that had been overlooked during the initial interpretation. This sequence highlights how early IAAA-associated infection can be radiologically subtle and easily misinterpreted as non-spinal pathology. Our case aligns with these reports and emphasizes the need for heightened clinical suspicion and evaluation of non-orthopedic causes in elderly patients with atypical spinal pain.

## Conclusions

In the present case, the absence of neurological findings and failure to perform an abdominal examination delayed the diagnosis. IAAA should be considered as a differential diagnosis in older males presenting with back pain, particularly when systemic signs of infection are present. Imaging can be misleading if soft tissue abnormalities are overlooked. Physicians must assess beyond bone and joint structures to avoid missing life-threatening conditions, such as IAAA. Careful review of imaging findings, including soft tissue and vascular structures, is essential in patients with atypical back pain.
